# Pulmonary atelectasis and survival in advanced non-small cell lung carcinoma

**DOI:** 10.3109/03009731003695624

**Published:** 2010-07-19

**Authors:** Yilmaz Bulbul, Bulent Eris, Asim Orem, Ayhan Gulsoy, Funda Oztuna, Tevfik Ozlu, Savas Ozsu

**Affiliations:** ^1^Department of Chest Diseases, Karadeniz Technical University, School of Medicine, TrabzonTurkey; ^2^Department of Biochemistry, Karadeniz Technical University, School of Medicine, TrabzonTurkey

**Keywords:** Atelectasis, NSCLC, obstructive pneumonitis, prognosis, survival

## Abstract

Atelectasis was reported as a favorable prognostic sign of pulmonary carcinoma; however, the underlying mechanism in those patients is not known. In this study, we aimed to investigate prospectively the potential impact of atelectasis and/or obstructive pneumonitis (AO) on survival and the relation between atelectasis and some laboratory blood parameters. The study was conducted on 87 advanced stage non-small cell lung cancer (NSCLC) patients. Clinical and laboratory parameters of patients at first presentation were recorded, and patients were divided into two groups according to the presence of AO in thorax computed tomography (CT). Survival was calculated using Kaplan-Meier and univariate Cox's regression analyses. Laboratory parameters that might be related with prolonged survival in atelectasis were compared using chi-square, Student's *t*, and Mann-Whitney *U* tests. Of the patients, 54% had stage IV disease, and AO was detected in 48.3% of all cases. Overall median survival was 13.2 months for all cases, 10.9 months for patients without AO, and 13.9 months for patients with AO (*P* = 0.067). Survival was significantly longer in stage III patients with AO (14.5 months versus 9.2 months, *P* = 0.032), but not in stage IV patients. Patients with AO in stage III had significantly lower platelet counts (*P* = 0.032) and blood sedimentation rates than did those with no AO (*P* = 0.045). We concluded that atelectasis and/or obstructive pneumonitis was associated with prolonged survival in locally advanced NSCLC. There was also a clear association between atelectasis and/or obstructive pneumonitis and platelets and blood sedimentation rate.

## Introduction

Despite the emerging developments in the diagnosis and treatment of lung cancer in recent years, it is still the most lethal among all cancers. In order to understand the pathobiology of lung cancer and also to predict survival better, several prognostic factors have been investigated in several studies (1). In a previous retrospective study, we showed that atelectasis was associated with prolonged survival (2). A recent study by Dediu and co-workers also focused on the favorable prognostic significance of atelectasis in non-small cell lung cancer (NSCLC) and pointed out the role of atelectasis in TNM staging (3). Indeed, atelectasis is accepted as a negative prognostic sign in the previous and recent TNM staging systems (4,5). Of the patients with lung cancer, about one-third to one-quarter are reported to have atelectasis at first presentation (2,6). In this study we aimed to investigate prospectively the potential impact of atelectasis and/or obstructive pneumonitis on survival and to investigate possible underlying conditions that might be related to prolonged survival in non-small cell lung cancer.

## Materials and methods

### Study design

This was a prospective observational study evaluating the role of atelectasis on survival and some related underlying conditions in NSCLC patients. The study was conducted at Farabi Hospital, a tertiary care hospital at Karadeniz Technical University, Turkey, and was approved by the local ethics committee.

### Study setting and population

All patients with NSCLC diagnosed and followed up in the chest clinic of Farabi Hospital between April 2006 and May 2008 and who met the inclusion criteria were enrolled in the study. Patients who gave written informed consent and had a performance status between 0–2 according to ECOG (European Cooperative Oncology Group) were included. Patients who had resectable lung cancer, who received prior chemotherapy or radiotherapy, who had no definitive histologic diagnosis, who had bad performance status (ECOG 3 or 4), and who had a disease other than lung cancer that might affect survival or the results of blood tests, such as liver cirrhosis, severe chronic obstructive pulmonary disease (COPD) or cardiac insufficiency, concomitant malignancy, etc., were excluded.

Patients were staged according to the TNM system. As part of routine clinical evaluation in our unit, staging was done using thorax, abdomen, and cranial tomography and bone scanning. Routine blood tests (biochemistry and complete blood counts) were done in all cases before each cycle of chemotherapy. In the treatment of patients, platinum-based treatment regimes (docetaxel, vinorelbine, or gemcitabine) were used every 3 weeks. Evaluation of treatment responses was carried out after three cycles of chemotherapy using Response Evaluation Criteria In Solid Tumors (RECIST) criteria (7). Following three cycles of therapy, external radiotherapy with a total dose of 63 Gy in 35 fractions was applied in locally advanced cases. Diagnosis of atelectasis and/or obstructive pneumonitis was confirmed using thorax computed tomography (CT) on first presentation. Blood test results obtained before the first cycle of chemotherapy were used for the comparison between patients with and without atelectasis.

### Statistical analysis

Survival was studied from the date of histopathologic diagnosis to the last contact with the patient. Survival curves were calculated using the Kaplan-Meier method, and statistical comparisons were performed by log-rank test. The relationship between atelectasis and survival was examined using univariate Cox's regression analysis. For the comparison of findings between patient subgroups, the chi-square test was used for quantitative data and Mann Whitney *U* or Student's *t* tests were used for qualitative data, and *P*-values < 0.05 were considered to be significant.

## Results

During the study period, 155 lung cancer patients were diagnosed and followed up in our clinic, and among those patients 87 NSCLC patients were included in the study; 47 with stage IV disease, 40 with locally advanced stage III disease (8 patients with stage IIIA, 32 patients with stage IIIB). Of the excluded patients, 28 had small cell lung carcinoma, 19 had resectable lung cancer, 13 had bad performance status (ECOG > 2), and 8 had not given signed informed consent or had concomitant diseases, etc. Of the cases included, the average age was 64.95 ± 10.56 years, and only 5 were women. Of all patients, 32 (36.8%) had only atelectasis (2 total, 16 lobar, and 14 segmentary) and 42 (48.3%) had atelectasis and obstructive pneumonia. Demographic characteristics of patients with and without atelectasis are given in Table I.

**Table I.** Demographic characteristics of patients with NSCLC.

	Patients with		
	AO (*n* = 42)	No AO (*n* = 45)	*P*
Age	64.09 ± 10.75	65.77 ± 10.44	0.465
Sex (Female)	3 (7.1%)	2 (4.4%)	0.669
Tumor size			
T1–2	4 (9.5%)	4 (8.9%)	NS
T3	9 (21.5%)	9 (20.0%)	
T4	29 (69.0%)	32 (71.1%)	
Lymph node involvement			
N0–1	7 (16.7%)	11 (24.5%)	NS
N2	30 (71.4%)	22 (48.9%)	
N3	5 (11.9%)	12 (26.6%)	
Metastasis (M1)	25 (59.5%)	22 (48.9%)	0.320
Cranial metastasis	5/42 (11.9%)	3/45 (6.8%)	0.479
Chemotherapy regimen (Platinum+)			
Docetaxel	28 (66.7%)	29 (64.4%)	NS
Gemcitabine	8 (19.0%)	7 (15.6%)	
Vinorelbine	4 (9.5%)	5 (11.1%)	
Other	2 (4.8%)	4 (8.9%)	
Performance status (ECOG)			
0	11 (26.2%)	11 (24.5%)	NS
1	28 (66.7%)	29 (64.4%)	
2	3 (7.1%)	5 (11.1%)	

NSCLC: Non-small cell lung carcinoma, AO: Atelectasis and/or obstructive pneumonitis, ECOG: Eastern Cooperative Oncology Group.

### Relationship between atelectasis and survival

Overall median survival was 13.2 months (range 0.2–32.0 months) for all cases, 10.9 months for patients with no atelectasis, and 13.9 months for patients with atelectasis (*P* = 0.067). When patients are discriminated as local and metastatic disease, survival was found to be 14.5 months with atelectasis in locally advanced patients and 9.2 months without atelectasis (*P* = 0.032). In univariate Cox's regression analysis, atelectasis was also associated with prolonged survival (odds ratio (OR) 2.43, 95% confidence interval (CI) 1.06–5.58, *P* = 0.037). However, in metastatic patients, survival was not different between atelectatic and non-atelectatic patients (Table II).

**Table II.** One-year survival rates and median survival in NSCLC patients with and without atelectasis.

	Patients with AO			Patients with no AO			
	1-year survival (%)	MST (months)	Range (months)	1-year survival (%)	MST (months)	Range (months)	*P*
Locally advanced cases	67.2	14.5	1.9–25.0	40.0	9.8	0.8–17.6	0.032
Stage IIIB	72.2	13.9	9.2–18.7	35.8	9.3	5.4–13.1	0.044
Metastatic cases	60.2	13.3	0.7–32.0	52.5	12.9	0.2–19.0	0.621
All cases	63.2	13.9	0.7–32.0	44.4	11.0	0.2–19.0	0.067

NSCLC: Non-small cell lung carcinoma, AO: Atelectasis and/or obstructive pneumonitis, MST: Median survival time.

Subgroup analysis of patients with locally advanced stage showed that survival was longer in stage IIIB patients with atelectasis (1-year survival rates and median survival in atelectatic patients were 72.2% and 13.93 months; in non-atelectatic patients they were 35.8% and 9.26 months, respectively, *P* = 0.044). However, because there were only 8 patients in stage IIIA, survival curves could not be calculated in this subgroup.

### Blood tests in patients with/without atelectasis

When locally advanced patients were taken into consideration, we found no difference in C-reactive protein (CRP), lactate dehydrogenase, alkaline phosphatase, hemoglobin levels, white blood cell counts, and lymphocyte counts in atelectatic and non-atelectatic patients (data not shown). However, platelet counts and blood sedimentation rates were significantly lower in patients with atelectasis. Platelet counts were 322.100 ± 90.100 cells/μL in the atelectatic group and 384.600 ± 80.100 cells/μL in the non-atelectatic group (*P* = 0.032). Blood sedimentation rates were 39 ± 17 mm/h in atelectatic and 53 ± 23 mm/h in non-atelectatic patients (*P* = 0.045).

## Discussion

The results of our study show that atelectasis was associated with prolonged survival, especially in locally advanced lung cancer. This finding was in accordance with two previous studies. In the first study, which included both small cell lung cancer (SCLC) and NSCLC patients, it was shown that atelectasis was associated with a prolonged survival especially in advanced stage lung cancer patients (2). In the recent study by Dediu et al. it was reported that patients with atelectasis had a favorable prognosis mainly in locally advanced NSCLC (3). In contrast to these data, only Coen at al. reported that survival was not different in patients with atelectasis (8). The effect of atelectasis was also investigated in stage IB tumors; however, it has been shown that presence of atelectasis in those patients has no impact on survival (9,10).

Atelectasis is a common condition with lung cancer. In our study, 36.8% of patients had atelectasis, and 48.3% had atelectasis and obstructive pneumonitis. Those results are in accordance with previous findings (2,6,11). In pulmonary carcinoma, atelectasis is usually developed due to endobronchial obstruction but less frequently due to compression of tumor or pleural effusion. It is known that vascular shunts develop in atelectatic areas (12). In our previous study, we had pointed out that prolonged survival in atelectasis might be related to decreased intratumoral blood flow and nutrition due to shunts in the neighboring atelectatic area (2). Actually, lower levels of platelet counts in patients with atelectasis might support this hypothesis. The relation between survival and platelets was studied in several studies, and thrombocytosis was reported to be related to poor prognosis (13–17). The mechanism underlying thrombocytosis is unclear; however, it is speculated to be related to the stimulation of bone-marrow by the cytokines (Interleukin (IL)-1, IL-6, and Macrophage-Colony Stimulating Factor (M-CSF)) secreted from cancer cells (18,19). If this is correct, decreased blood supply and nutrition of tumor due to atelectasis and shunt effects may decrease the release of these cytokines from cancer cells. In addition, patients without atelectasis had increased blood sedimentation rates than did atelectatic patients. The increased sedimentation rate in non-atelectatic patients may be related to increased fibrinogen synthesis by well nourished cancer cells. Sahni et al. reported that fibrinogen synthesized from cancer cells may promote the growth of lung and prostate cancer cells through interaction with fibroblast growth factor-2 (FGF-2) (20).

Another hypothesis that might relate to prolonged survival is the susceptibly of infection and the altered immunity in atelectasis. Nguyen at al. reported that natural killer and lectin-dependent cell-mediated cytotoxicity activities in peripheral blood lymphocytes were significantly increased in animals with right lower lobe atelectasis (21). They also reported that atelectasis was associated with an influx of polymorphonuclear leukocytes into the bronchoalveolar compartment. However, in our study, in locally advanced cases, we found no correlation between atelectasis and CRP, a non-specific marker of systemic inflammation. In addition, there was no correlation between atelectasis and white blood cell counts and lymphocytes. The negative prognostic significance of high CRP levels and peripheral blood leukocyte count in pulmonary carcinoma has been reported in some prior studies (18,19,22,23).

Dediu et al. claimed that prolonged survival in atelectasis was associated with the specific growing pattern of tumor (3). According to this hypothesis, patients with atelectasis present earlier and receive earlier diagnosis due to concentric growth of the tumor. However, we think the similarity of T, N, and M status between patients with and without atelectasis on first admission does not support this hypothesis.

We acknowledge some restrictions of this study. First of all, the number of patients included in the study is limited. Also, the effect of atelectasis and shunts on the nutrition of tumor tissue would be better shown if we had measured some cytokines and hormones secreted by the cancer cells. Lastly, we did not consider patients who developed atelectasis after the first presentation, i.e. during follow-up. Because of these limitations, a complete analysis of all factors that may be related to atelectasis and prognosis should be considered in a prospective study.

In conclusion, our study has shown that atelectasis and/or obstructive pneumonitis was associated with prolonged survival in locally advanced NSCLC cases. We also found a significant correlation between atelectasis and platelets and blood sedimentation rate. Although the relationship between atelectasis and survival was in accordance with two previous studies, we think more comprehensive studies are needed to reach a final judgment on the effect of atelectasis on survival.
